# Implementing Web-Based Therapy in Routine Mental Health Care: Systematic Review of Health Professionals’ Perspectives

**DOI:** 10.2196/17362

**Published:** 2020-07-23

**Authors:** Fiona Davies, Heather L Shepherd, Lisa Beatty, Brodie Clark, Phyllis Butow, Joanne Shaw

**Affiliations:** 1 Psycho-Oncology Co-operative Research Group School of Psychology The University of Sydney Sydney Australia; 2 Flinders Centre for Innovation in Cancer College of Medicine & Public Health Flinders University South Australia Adelaide Australia

**Keywords:** health professional views, implementation, online psychological therapy, online CBT, barriers, facilitators, models of care, cognitive behavioral therapy, internet-based intervention

## Abstract

**Background:**

Web-based therapies hold great promise to increase accessibility and reduce costs of delivering mental health care; however, uptake in routine settings has been low.

**Objective:**

Our objective in this review was to summarize what is known about health care professionals’ perceptions of the barriers to and facilitators of the implementation of web-based psychological treatments in routine care of adults in health care settings.

**Methods:**

We searched 5 major databases (MEDLINE, EMBASE, PsycINFO, CINAHL, and the Cochrane Library) for qualitative, quantitative, or mixed-methods studies exploring health professionals’ views on computer- or internet-based psychological treatment programs. We coded included articles for risk of bias and extracted data using a prepiloted extraction sheet.

**Results:**

We identified 29 eligible articles: 14 qualitative, 11 quantitative, and 4 mixed methods. We identified the following themes: patient factors, health professional factors, the therapeutic relationship, therapy factors, organizational and system factors, and models of care. Health professionals supported web-based therapies only for patients with relatively straightforward, low-risk diagnoses, strong motivation and engagement, high computer literacy and access, and low need for tailored content. They perceived flexibility with timing and location as advantages of web-based therapy, but preferred blended therapy to facilitate rapport and allow active monitoring and follow-up of patients. They emphasized the need for targeted training and organizational support to manage changed workflows. Health professionals were concerned about the confidentiality and security of client data for web-based programs, suggesting that clear and transparent protocols need to be in place to reassure health professionals before they will be willing to refer.

**Conclusions:**

Without health professionals’ support, many people will not access web-based therapies. To increase uptake, it is important to ensure that health professionals receive education, familiarization, and training to support them in incorporating web-based therapies into their practice, and to design systems that support health professionals in this new way of working with patients and addressing their concerns.

**Trial Registration:**

PROSPERO CRD42018100869; https://tinyurl.com/y5vaoqsk

## Introduction

### Background

Internationally there is a move toward using digital technologies in mental health care, including the development of an increasing number of Web-based therapy programs [1,2]. The reasons for this interest in electronic mental health commonly include accessibility, flexibility (in terms of when and where they can be accessed), lack of mental health care professionals to cover need, and cost considerations [3,4]. Randomized controlled trials of web-based therapies show promise, with efficacy reported to be similar to that of face-to-face interventions [5]. A systematic review of internet-based cognitive behavioral therapy [6], for example, found that this therapy has been tested for 25 different clinical disorders, with large effect sizes meeting the highest level of criteria for evidence for disorders such as depression, anxiety, and severe health anxiety.

Despite this interest, research on referral and uptake of web-based therapies in routine care is limited [7]. In a rapid review of literature on this topic, we found no studies set in hospital-based medical clinics and only 1 study in primary care, which reported extremely low rates of referral (14% of the expected number) and uptake (1% of those referred) [8]. A major implementation project in Europe, known as MasterMind, is studying web-based therapies in routine mental health care [9,10], but no data are published at this stage.

Nor is there a large body of evidence on effective processes for implementation of web-based therapies in routine clinical care [7]. A recent review of staff-reported barriers to and facilitators of hospital-based interventions of any kind (web-based or face-to-face) [11] identified 3 key domains that may affect implementation success—system (eg, workload and workflow, physical structure and resources, culture, communication, and external pressures), staff (eg, attitudes, understanding and awareness, role identity, skills, and confidence), and intervention (eg, ease of integration, validity and evidence base, safety and ethics, supporting resources)—with similar barriers likely in community settings [12]. Several conceptual frameworks have been developed to further explicate and guide health care implementation, including the Consolidated Framework for Implementation Research (CFIR) [13]. CFIR is composed of 5 major domains: intervention characteristics, the inner setting, the outer setting, characteristics of individuals involved in implementation, and the implementation process. As health professionals often act as gatekeepers to web-based programs, they are key stakeholders and may be very influential in determining whether web-based programs are disseminated widely. Thus, in this systematic review, we decided to focus on health professionals’ perspectives of web-based therapies, which falls within the fourth CFIR domain.

### Objective

Several individual studies reported data on health professionals’ perceptions of web-based therapy programs, which revealed concerns about therapeutic alliance and the quality and effectiveness of programs [14,15], patient commitment and compliance [15,16], patient barriers such as internet literacy and access [16,17], and suicide risk [16]. However, we found no synthesis of these studies to provide an overall picture of health professionals’ perspectives to date. Thus, we sought to fill this gap by conducting a systematic review. Our research question was what are health professionals’ perceptions of the barriers to and facilitators of the implementation of web-based psychological treatments in routine care in health settings?

## Methods

### Review Registration

We registered the review with the International Prospective Register of Systematic Reviews (PROSPERO CRD42018100869).

### Searches

We searched 5 bibliographic databases: MEDLINE, EMBASE, PsycINFO, CINAHL, and the Cochrane Library (Cochrane Database of Systematic Reviews, Cochrane Central Register of Controlled Trials, Cochrane Methodology Register). The search strategy included terms relating to web-based approaches, psychological therapies, and views of barriers to and facilitators of dissemination.

Qualitative, quantitative, or mixed-methods studies exploring health professionals’ views on computer- or internet-based psychological treatment programs were eligible if the treatment contained a component designed to change psychological symptoms or behaviors either associated with a diagnosable mental health condition (using *International Classification of Diseases, Tenth Revision*, Diagnostic and Statistical Manual of Mental Disorders [Fifth Edition], or equivalent) or secondary to a physical health condition such as cancer. This could include anxiety, depression, substance misuse, suicidality, insomnia, complex pain, fatigue (eg, chronic fatigue syndrome or cancer-related fatigue), or medication adherence. Studies of views on all models of web-based therapy, from entirely self-directed to blended with face-to-face care, were eligible. Additionally, the patients targeted (and the health professionals) could be situated either in a hospital or clinic setting or in the general community.

We included only studies investigating views on web-based programs for adults (>18 years of age); we excluded programs targeting adults who were completing the program on behalf of someone else, for example, parents completing a program to help their children with anxiety or behavioral problems.

We included studies of the views of all health professionals involved in referring, triaging, or providing psychosocial care to patients with the above conditions (eg, doctors, nurses, psychosocial health professionals, allied health practitioners). We excluded nonclinical professional groups who do the above (eg, schoolteachers, religious advisers).

We limited included studies to peer-reviewed articles, excluding conference abstracts. We searched studies published from 1986 (when the first known digital mental health site, Ask Uncle Ezra, at Cornell University was launched) to the end of October 2019.

Titles or abstracts, or both, of articles retrieved during the search strategy or from reference lists were screened by 1 reviewer to identify studies that met the inclusion criteria described above. As an initial step, 10% of the titles or abstracts and full-text articles were independently reviewed by a second reviewer to confirm inclusion and exclusion decisions. Following this, any titles or abstracts or full-text articles where the reviewer had any uncertainty about the decision was put aside and discussed with a second reviewer. Any continuing uncertainty was resolved through retrieving the full text of the study or discussion with other members of the authorship team. We hand searched reference lists of included articles for relevant articles.

### Data Extraction

We extracted information from articles meeting the inclusion criteria, with 2 team members completing extraction for each article. Any uncertainty was resolved through discussion with additional members of the review team. Data extracted were author, country, study design, sample characteristics, web-based therapy characteristics, measures, results, and limitations.

### Risk-of-Bias (Quality) Assessment

We assessed the selected articles for risk of bias using checklists appropriate to quantitative and qualitative studies from Kmet et al [18] and to mixed-methods studies from Pluye et al [19]. All articles were graded by 2 team members and discussed with the author team if there was any ambiguity in grading.

### Strategy for Data Synthesis

We analyzed the articles using a 3-stage synthesis process that combined qualitative, quantitative, and mixed-methods studies comprehensively and rigorously to answer the review question, following the methods of Thomas et al [20]. First, we summarized quantitative data using descriptive statistics. Second, we synthesized qualitative data by themes [21]. All authors coded 2 qualitative studies line by line to develop a draft coding tree and themes, which we then refined after coding 2 additional studies. We then applied this coding structure to the remaining qualitative studies, with emerging themes created and discussed by all authors. Similarities and differences between codes and their content were examined to develop higher-order themes. (See Multimedia Appendix 1 for the final coding tree.) Third, we used a matrix to compare the qualitative, quantitative, and mixed-methods findings from the first 2 stages to answer the review research question.

## Results

### Search Results

**Figure 1:**
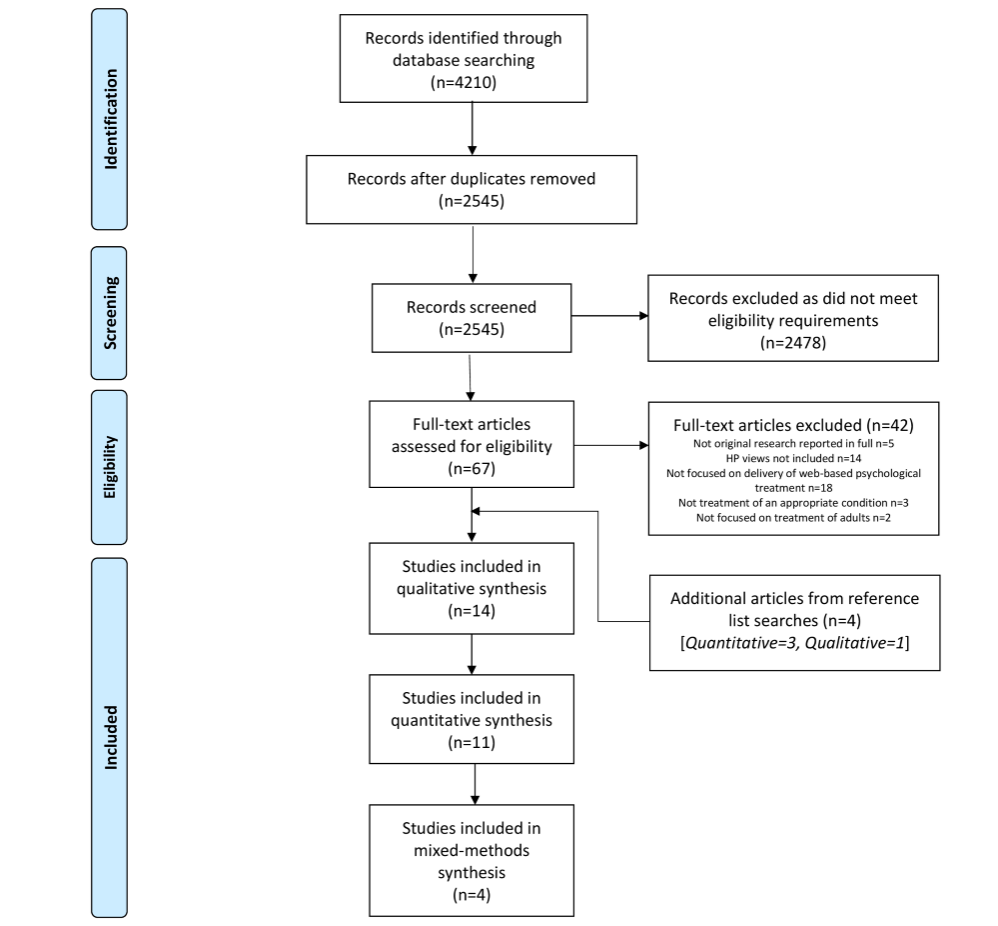
Preferred Reporting Items for Systematic Reviews and Meta-Analyses (PRISMA) flow diagram. HP: health professional.

We initially identified 4210 potentially eligible articles from the search, with 2545 remaining after deduplication. Figure 1 shows the Preferred Reporting Items for Systematic Reviews and Meta-Analyses (PRISMA) flow diagram, with 29 articles meeting full inclusion criteria: 14 qualitative, 11 quantitative, and 4 mixed methods. Most articles were published since 2009 and reported results from the United Kingdom (n=5), the United States (n=6), Australia (n=4), Canada (n=4), Europe (n=10), and Israel (n=1). A total of 16 studies specified the professional background of the included health professionals (psychologists, n=12 studies; social workers, n=9 studies; general practitioners, n=4 studies; nurses, n=3 studies; and psychiatrists, n=2 studies), and the remaining 13 studies used general terms (eg, “psychotherapist” or “counsellor” or “practitioner”). Where studies included both health professionals and patients, we included only data from the health professionals in the review. See Multimedia Appendix 2 for study characteristics. All included articles were of acceptable quality, with reflexivity and use of verification procedures to establish credibility the most poorly done (see Multimedia Appendix 3 for quantitative studies, Multimedia Appendix 4 for qualitative studies, and Multimedia Appendix 5 for mixed-methods studies).

### Thematic Synthesis

We identified 6 themes, which mapped well to the CFIR: therapy factors, organizational (or inner setting) and system (or outer setting) factors, patient factors, health professional factors, the therapeutic relationship, and models of care.

#### Therapy Factors

In total, 3 of 11 quantitative studies (n=677) [22-24], 5 of 14 qualitative studies (n=78) [14,25-28], and 3 of 4 mixed-methods studies (n=111) [17,29,30] explored the impact of therapy factors on the uptake of web-based therapies. Findings suggested that health professionals believed there was evidence [29,30] supporting the utility of web-based therapies but were generally more comfortable using web technologies as an *adjunct* to therapy, rather than as a *stand-alone* intervention [17,24,26] (see Models of Care subsection below for more detail). Overall, health professionals were neutral to positive toward web-based programs and identified the following consistent therapeutic factors that facilitate or hinder web-based therapy uptake.

The therapeutic content and approach of therapies were frequently raised by study participants as being more or less appropriate for a web-based format, with therapists more likely to refer patients for web-based psychoeducation, monitoring, and skills practice [17,23,26] within a positive psychology, mindfulness, acceptance and commitment therapy, or cognitive behavioral therapy (CBT) framework [17,23]. They were less likely to consider web-based therapy appropriate for psychoanalytic, interpersonal psychotherapy, or schema therapies. Nor were they comfortable with serious gaming [17,22], chat, or video components [17,24]. Concerns were raised regarding privacy of data, ethical considerations for suicidal patients, and levels of evidence available for intervention effectiveness [22,23].

The structured nature of web-based programs, the most commonly raised theme, was extensively discussed as both a facilitator of and barrier to uptake or recommendation. Whereas structure provided focus and aided navigation of programs [27,28], other studies noted that this needs to be balanced with flexible tailoring of content [29,30], a challenge that is inadequately addressed [14,27]. Some health professionals in the qualitative studies reported wanting to see more transdiagnostic options [25]. Hadjistavropoulos and colleagues partially addressed this recommendation in their mixed-methods study of a transdiagnostic web-based CBT intervention, with feedback specifically citing the transdiagnostic nature as a strength of the program [29].

#### Organizational and System Factors

A total of 6 of 11 quantitative studies (n=4171) [23,24,31-34], 10 of 14 qualitative studies (n=158) [14,25-28,35-39], and 2 of 4 mixed-methods studies (n=99) [17,29] reported organizational and system concerns, which fall within the second and third domains of the CFIR.

##### Training

Training was highlighted as driving confidence in recommending web-based therapy options to patients [28]. Health professionals wanted more information, training, clear guidelines, and information on liability to be comfortable with web-based approaches [34].

Training to improve computer skills and stay up to date [23] and the need for better information technology support were also identified [17]. Qualitative studies [14,25,28] highlighted training needs in email or electronic communication skills in a therapeutic context [14,25] and, from an organizational perspective, the time required to obtain skills and to respond to patients in this format. Notably, 1 study suggested that the opportunity to pause and reflect before responding to emails was a strength of web-based therapy [37].

##### Time Saver or Time Changer

Health professionals reported varying views regarding whether web-based approaches made therapy delivery harder or easier. In the study by Bengtsson et al [27], therapists who were experienced in delivering both web-based and face-to-face therapy noted that work time was more flexible with web-based CBT, enabling work during cancellations, and flexibility in appointment times and place of work. Other advantages included therapy being unaffected by illness, emergencies, or other work; allowing colleagues to take over each other’s work if needed; and the potential to work with more clients at once [27]. In contrast, another study noted that web-based therapy was time consuming for both patients and therapists, with experienced web-based therapists spending more time overall on web-based treatment than with face-to-face treatment [25].

##### Managerial and Organizational Support

In 2 studies, support and leadership from organization staff and local management was noted as valuable in encouraging use of web-based CBT [17,25]. In another quantitative study [31], perceived supportiveness of the organizational environment predicted intention to use web-based CBT. A large study found small effects of workplace factors, such that working in a community treatment center (rather than private practice) and having good access to technology at work increased perceptions of the efficacy of blended treatment [32].

##### Accessibility and Integration

Some studies reported that health professionals found it easier to integrate written materials into care, rather than referring to web-based programs where specific content is difficult to access easily [38,39]. In the study by Batka and colleagues, health professionals expressed mixed views regarding the ability of web-based therapy to bridge the gap between primary care and behavioral health [36]. One study supported the idea of web-based therapies identifying those in need of additional support [35]. Several studies noted the need to supplement patient self-referral and management with health professional monitoring and follow-up, to ensure the program matched the patient’s needs [14,28] and to encourage adherence. Rural health professionals in 1 study [26] expressed concern that an increase in web-based treatment may further isolate rural patients without internet access:

Often online services have been looked at as, you know, the great hope for areas where there aren’t real services for people, but where there isn’t adequate internet access, you are further marginalizing people who live in remote areas who now don’t have access to two different services...

pg 5

##### Data Security and Privacy

In 1 study, confidentiality of patient information was listed as the primary concern by 48% of health professionals, with 81% stating they had some concern [33]. Two quantitative studies highlighted health professionals’ concerns about information security [17,23], ethical or clinical guidelines [17,23], and legal issues or liability [17], before supporting use of web-based therapies. Concern about confidentiality was raised in 1 study, where health professionals expressed preferences for using secure email or an app over chat or video formats [24]. Qualitative studies identified protocols to address privacy and ethical practice in an information technology context [37,38].

#### Patient Factors

A total of 4 of 11 quantitative studies [22,23,33,34] incorporating data from 3144 health professionals, 9 of 14 qualitative studies (n=158) [25,26,35-38,40-42], and 3 of 4 mixed-methods studies (n=374) [29,30,43] raised issues related to patient factors. Most health professionals in a quantitative study [22] believed that web-based therapies would be appropriate for 20% of patients; health professionals in another study perceived barriers in 22% to 48% of patients [23]. One study argued that web-based therapies allowed patients to become active participants in their own recovery [40].

##### Nature and Severity of Symptoms

Patient factors identified in both quantitative and qualitative studies included the nature and severity of symptoms, with anxiety disorders commonly cited as a good fit for web-based therapy [26,34,43], and more complex or severe disorders viewed as a poor fit [26,37,40]. As anxiety and depression symptom severity increased from mild to severe, the disorders were seen as having decreasing fit for web-based therapy [34].

##### Individual Characteristics

Health professionals were concerned that comorbidity [25] or suicidal ideation required risk management [35]. It was also noted [30] that web-based therapy cannot easily take into account patient characteristics including comorbidity, needs and motivation, written expression, skills, and personality (including self-management). For example [37]:

I found for my anxiety clients, it worked quite well. For the depression clients, who are more severe, I found that they tend to take much longer...there’s less motivation...those clients, maybe it would be better for them to like see somebody in person because there’s a lot of other issues.

pg 44

##### Accessibility

Health professionals identified access to face-to-face treatments as a factor, with several studies noting that web-based therapies were particularly attractive and useful to patients who could *not* readily access therapy [30] (eg, those with limited mobility, time, or access to local services [37,38], with perceived stigma [29], or in rural or remote locations [33]).

##### Practical Barriers

Health professionals expressed concern about practical barriers such as access to technology [23] or the internet [35,41]. Additional barriers included having poor literacy, not being “psychologically minded,” or having difficulties with vision or hearing [41]. Health professionals commented that their patients often had grade 6 to 8 reading levels and could not cope with the reading required [36]. This could also impact ability to complete written tasks [37]:

If they don’t provide the information or if they’re having trouble expressing exactly what the problem was, then it’s obviously going to be more difficult.

pg 46

#### Health Professional Factors

Health professional factors relevant to the uptake of web-based therapies were raised in 9 of 11 quantitative studies (n=4536) [22,23,31-33,44-47], 6 of 14 qualitative studies (n=96) [25-28,38,48], and 3 of 4 mixed-methods studies (n=407) [17,30,43]. Quantitative studies raised few health professional issues. A total of 3 studies suggested that psychodynamic therapists are less positive toward web-based therapy [22,32,46], but another found no difference [44]. One study found that openness to evidence-based practice predicted more favorable attitudes [32], whereas another found that expectations of ease of use predicted intentions to use web-based CBT [31]. The main issue raised was lack of familiarity with web-based therapy, consistent with the low uptake of these approaches generally [17,23,31,45,47].

Several barriers related to health professionals were raised in the qualitative studies. There were concerns about the technological skills and comfort level of health professionals [26,48]. Significant concerns were raised about workload issues: finding time to incorporate web-based treatment [28] and managing the different demands of electronic work [25]. A final factor raised by therapists was that they saw web-based treatment as both less engaging and less taxing than face-to-face treatment. For example [27,38]:

I just felt like I was in a call centre rather than being a clinician working with patients who had difficulties.

pg 8

You feel very much less burdened by [internet-based CBT] than in regular outpatient care.

pg 473

The mixed-methods studies raised concerns related to health professionals’ knowledge and training in web-based therapy. Motivation and use of web-based therapy were low, with reported rates of 2% [33] and 2.4% [43], in part reflecting misunderstandings about confidentiality and liability [33]. For example [30]:

My concern is not to get patients motivated to use the online modules, but to get therapists to use them. That is a much larger bump. Everything that is new is seen as more work, and everybody is already loaded with work and doesn’t want more work. I’m afraid that pressure from management is the only way to get therapists to work with it.

pg 7

#### The Therapeutic Relationship

A total of 4 of 11 quantitative studies (n=3755) [22,23,32,33], 6 of 14 qualitative studies (n=751) [25,27,28,38,39,48], and no mixed-methods studies explored the impact of web-based therapy on the quality of the health professional-patient therapeutic relationship. Across studies, health professionals who were experienced in web-based therapy held more positive views of the potential for a therapeutic relationship in web-based therapy [33,38,39].

Quantitative findings highlighted minimal concern among health professionals about establishing and maintaining a therapeutic relationship through mobile apps [23] or blended face-to-face and web-based therapy, although concerns were raised in 1 study that including serious games may lead to neglect of relationships and communication during therapy [22]. Health professionals in another large quantitative study indicated that they believed integrating web-based approaches in therapy would interfere with rapport [32].

Among qualitative studies, health professionals perceived the therapeutic relationship as *different from that in face-to-face therapy but not necessarily worse*, and some reported being surprised by their ability to develop relationships online [25]. Bengtsson et al [27] highlighted that the web context may extend the time needed to develop a therapeutic relationship but, as it is less confronting, may be particularly helpful in building relationships with patients with social anxiety.

Two studies reported the views of primary care physicians [28,48], who were ambivalent about the impact of web-based therapy on therapeutic relationships, noting that psychodynamic approaches relied on *open and active dialogue* between health professional and patient.

#### Models of Care

A total of 3 of 11 quantitative studies (n=677) [22-24], 8 of 14 qualitative studies (n=129) [14,25,28,35,36,38,39,42], and all 4 mixed-methods studies (n=440) [17,29,30,45] explored health professionals’ views and preferences regarding models of care delivery incorporating web-based components. In addition, 9 studies [14,25,28,29,35,38,39,42,43] investigated use of web-based therapies as a stand-alone treatment, 2 studies [22,23] discussed preferences for serious gaming and mobile apps as an adjunct to therapy, and 3 studies [17,24,30] explored blending face-to-face and web-based components as part of an integrated approach to therapy. One study [36] included the views of mental health and primary care providers as 1 model of care delivery in a suite of telehealth options.

Among web-based therapy studies, views differed based on whether the therapy was guided or self-directed (low intensity). Although it was acknowledged that self-referral to self-help modules increased ease of access for patients [39] and made it more likely that patients were motivated to engage with treatment [14], there were concerns that this model of care did not provide sufficient health professional support, especially for challenging tasks [29,38]. Health professionals were more comfortable with guided self-help, as they perceived this as fitting more within the therapeutic role [38]. General practitioners trained to administer guided web-based CBT highlighted the challenges in integrating web-based therapy process issues into consultations [28]. Overall, there was a preference to use web-based therapy flexibly, possibly as an adjunct to face-to-face treatment, [38,42,43], as an option to support those on a waiting list for treatment [29] or as part of a stepped-care model [25].

Blended therapy was perceived as a viable alternative to a web-based model, as compatible with current clinical practice, and as raising few concerns about treatment efficacy with the inclusion of web-based components as part of a wider face-to-face model [17,24]. Models of care that incorporated serious gaming and use of mobile apps were perceived as potentially useful adjuncts to traditional therapy approaches to support skills development both during and after therapy [23,30].

## Discussion

### Principal Findings

Despite good evidence of efficacy in randomized controlled trials [5], the potential of web-based psychological therapies to increase access to mental health care has not been well realized in nontrial settings to date [49]. This is, to our knowledge, the first synthesis of quantitative and qualitative data on health professionals’ perceptions of web-based therapy for mental health conditions (both primary and secondary to physical disorders). We found that health professionals perceived many barriers to routine use of web-based therapy and had strong views about appropriate models of care. While health professionals’ concerns may not all be evidence based, health professionals are in many cases the gatekeepers to referral. Therefore, addressing their concerns is a key strategy to improve implementation, alongside ongoing education of health professionals so that they are familiar with the content, processes, and evidence base of web-based therapies and are comfortable with incorporating them into their model of care. The importance of thinking carefully about models of care specifically in the context of web-based therapies has not been addressed adequately in the literature to date.

Health professionals’ concerns corresponded closely with the 5 domains of the CFIR [13], with issues raised pertaining to intervention characteristics (eg, flexibility), the inner setting (eg, managerial support), the outer setting (eg, data security and privacy), characteristics of individuals (eg, their health and internet literacy), and the implementation process (eg, health professional education). Furthermore, they also accord with the much broader findings of a systematic review of reviews concerning factors that influence implementation of electronic health [50]. We found, as did the review of reviews, that the factors influencing implementation are multilevel and complex, with no single factor acting as a key barrier or facilitator.

This review suggests that for web-based treatments to maximize their acceptability to health professionals, they should incorporate the following features.

#### Consider Tailoring

Health professionals noted that the highly structured nature of many web-based therapies, while easier to navigate [27], may contribute to patients’ sense of being funnelled through a preset and rigid program [25,27]. This accords well with surveys of patients regarding reasons for dropout from web-based programs. For example, in a qualitative study of patients who had dropped out of a transdiagnostic web-based therapy program, they noted the lack of specificity of content to their own particular problems [51]. Indeed, dropout rates for web-based compared with face-to-face programs are reported to be 10% to 15% higher, reinforcing the need for careful consideration of tailoring, as well as treatment credibility and engaging content, which also predict dropout [52].

#### Target a Subset of Patients

Even successful online clinics do not see web-based therapy as suitable for all individuals [49], and patients also report a preference for face-to-face treatment over web based [53]. This was reflected in our findings, as health professionals supported the potential utility of web-based therapies for a subset of patients only [22,23]. The findings suggest that, to increase referrals and uptake, web-based treatments should be targeted toward individuals with mild to moderate anxiety and depression, with limited comorbidity and low risk. Further patient factors that may lead to more referrals include sufficient computer literacy and access, good motivation, good health literacy, needing more flexible access to treatment, and concerns about stigma.

#### Use an Approach That Blends Web-Based Therapy With Face-to-Face Therapist Contact

Health professionals reported concern about wholly self-directed treatments, with a clear preference for blended treatments including some therapist face-to-face contact, as it affords a better opportunity to establish rapport and allows active monitoring and follow-up of patients as they move through online tasks [29,38]. This is in line with patient research, which showed a preference for face-to-face treatment over web based [53], and that adherence increased with therapist support [54]. Whereas health professionals who have no experience with web-based treatments are concerned about the therapeutic alliance, those who were experienced in supported web-based treatment were much less concerned. This suggests that models of care that incorporate some level of therapist support are likely to become more acceptable over time as health professionals become accustomed to the model.

#### Select Therapeutic Approaches and Content That Are Appropriate for a Web-Based Format

Prioritizing psychoeducation, monitoring, and skills practice, with more complex or interpersonal interventions reserved for face-to-face treatment, may make health professionals more confident in web-based treatments. Considering the balance between providing a clear structure and yet allowing content to be tailored to individual needs may also be important, although our review does not offer any clear guidance on how to optimally achieve that.

#### Educate Health Professionals in Web-Based Treatment

The vast majority of health professionals are unfamiliar with web-based therapies [17,23,31,45,47], and this needs to be addressed in order to increase rates of referral and use. Implementation of any model of care would need to consider how to educate health professionals about web-based therapy, including how to make appropriate referral decisions, find high-quality evidence-based resources, and incorporate web-based treatment as part of routine care.

#### Design a Supportive System

The review suggests that the broader setting for health professionals is important in successful implementation. This has 4 aspects.

First, the effect of web-based therapy on the work of frontline mental health care workers would need to be carefully considered to ensure that therapists have adequately engaging work and appropriate supervision in skills specific to web-based therapy such as written communication. Providing web-based therapy requires a different workflow from face-to-face treatment, and this would need to be managed. Web-based therapy was seen as both less rewarding and less draining than face-to-face work; the former was a strong disincentive to some health professionals, who felt that their engagement in, and sense of reward from, delivering expert and successful care was being taken from them [27,38]. A sense of reward is a key dimension of stress and burnout in occupational health [55], and work would need to be designed in a way that is satisfying for health professionals.

Second, the provision of adequate technological resources and support is important, as well as good data security.

Third, clear policies and procedures are needed regarding confidentiality, risk management, and liability issues.

Fourth, clear support and expectations from management regarding the use of web-based therapy are required, including impact on workflow and referral pathways for face-to-face therapy.

### Limitations

This review identified only 29 articles examining health professionals’ perspectives on web-based therapy. These articles varied considerably in terms of the population of health professionals included as participants, as well as the model of care that was being examined (eg, blended, self-directed), and are unlikely to give a complete picture. The main limitation in the literature so far is the inclusion of a very small proportion of health professionals who are experienced in web-based therapy, with most health professionals responding to questions in the absence of direct experience.

### Comparison With Prior Work

This is, to our knowledge, the first systematic review of health professionals’ perspectives on web-based therapy and provides a synthesis of the research to date.

### Conclusions

The findings of this review echo the factors found in a recent article documenting the experiences of 2 successful online clinics [49], which identified the importance of considering the patient, therapists and their training, comprehensive organizational and systemic support, and the place of web-based therapies in models of care. These results have important implications for the implementation of web-based therapy, emphasizing that important preparatory work is required if implementation is to be successful, in the way the intervention is designed, the training and support health professionals receive, and the engagement of senior management in supporting the transition to a different model of care.

Further research is needed to examine the acceptability of specific models of care [2], which may vary in level of therapist support, timing of the intervention (waitlist, posttherapy, etc), positioning in stepped care, or the way it is incorporated in routine care. As health professionals gain experience with web-based therapies, it will be important for their perspectives to be sought again, to elicit perspectives grounded in real interactions and patient feedback. Interventions to educate and support health professionals in using web-based methods will need to be developed and evaluated. Finally, large-scale implementation studies that document the implementation strategies employed, uptake and retention rates, and effectiveness outcomes will be needed to provide a solid evidence base for this therapeutic approach moving forward.

## Appendix

Multimedia Appendix 1 Coding tree.

Multimedia Appendix 2 Study characteristics.

Multimedia Appendix 3 Risk-of-bias assessment for quantitative studies.

Multimedia Appendix 4 Risk-of-bias assessment for qualitative studies.

Multimedia Appendix 5 Risk-of-bias assessment for mixed-methods studies.

## Abbreviations

CBT: cognitive behavioral therapy
CFIR: Consolidated Framework for Implementation Research
