# Supplementary material for: Implementing Web-Based Therapy in Routine Mental Health Care: Systematic Review of Health Professionals’ Perspectives
Source: J Med Internet Res. 2020 Jul 23;22(7):e17362. doi: 10.2196/17362 (PMC7413287; doi:10.2196/17362)
Supplement: Multimedia Appendix 1 [file jmir_v22i7e17362_app1.docx]

| **Major Theme** | **Codes** |
| --- | --- |
| Therapy Factors | - Content - Type of therapy - Format of therapy - Structure vs tailoring - Transdiagnostic approaches - Risk management |
| Organisational and System Factors | - Training - Workload and workflow - Support for health professionals - Accessibility for consumers - Integration with existing care - Data security and privacy - Ethical/clinical concerns - Legal issues/liability |
| Consumer Factors | - Suitable for a subset of consumers - Nature and severity of symptoms - Comorbidity and suicidal ideation - Individual characteristics (e.g., motivation, written expression) - Access to face-to-face treatments - Practical barriers (e.g., internet access, vision or hearing difficulties) |
| Health Professional Factors | - Familiarity with online therapy - Therapeutic orientation - Comfort with technology - Workload - Engagement and work satisfaction - Concerns about confidentiality and liability |
| Therapeutic Relationship | - Rapport - A different relationship |
| Models of Care | - Self-referral - Guided therapy - Blended care - Flexibility in use - Place in clinical pathway (e.g., waitlist, stepped care) |
