# Supplementary material for: Implementing Web-Based Therapy in Routine Mental Health Care: Systematic Review of Health Professionals’ Perspectives
Source: J Med Internet Res. 2020 Jul 23;22(7):e17362. doi: 10.2196/17362 (PMC7413287; doi:10.2196/17362)
Supplement: Multimedia Appendix 2 [file jmir_v22i7e17362_app2.docx]

| **Source** | **Country** | **Setting** | **Definition of online** | **Data Collection Methodology** | **Sample size** | **Age (Mean & range)** | **Gender** | **Profession** | **Role** | **Summary of results** |
| --- | --- | --- | --- | --- | --- | --- | --- | --- | --- | --- |
| Advocat & Lindsay 2010^1^ | Australia | Internet-based RCT, university based. | Online CBT for panic disorder with email support | Qualitative interviews and observations of meetings | 8 | Not reported | Not reported | Clinical Research Psychologists | Trialists in RCT | Researchers saw “good consumers” and “good participants” as the same. Consumers saw this differently with a tension between taking an active role and complying with the treatment. |
| Alberts et al. 2018^1^ | Canada | Cancer centres | 5 online CBT sessions. Therapist had weekly, regular contact via phone or email till completion. | Qualitative interviews after viewing a video about the online CBT program | 10 | 46.3 (31-60) | 10/10 female (100%) | 10/10 social workers (100%) | Social worker in cancer centre | Identification of barriers and facilitators from a provider perspective |
| Batka et al. 2016^1^ | USA | Primary care clinics in the US army participating in a large RCT to assess effectiveness of an enhanced collaborative care approach for PTSD and depression. | Online therapy as adjunct to other therapies | Qualitative interviews | 31 care providers and 7 care facilitators | Not reported | Not reported | Care providers: 18/31 Mental health (58%); 13/31 Primary care (42%)  Care facilitators: 7 nurses | Providing care to US army soldiers | Participants viewed collaborative care as another pathway for care but varied in how they thought this would work and which tools were best. |
| Becker & Jensen-Doss 2013 | USA | National survey, varied settings | Computerised therapies implemented with therapist support | Quantitative mailout survey | 1067 | 58.2 (28-85) | 304 males (28.5%), 745 females (69.8%) | 390 counsellors (36.6%), 304 social workers (28.5%), 373 marriage and family therapists (35.0%) | Mental health clinicians, 639 (59.8%) in private practice | Therapists were generally positive toward online therapies, but willingness or ability to use them varied across individuals. |
| Bengtsson et al. 2015 | Sweden | Psychotherapists who had worked with CBT online and face-to-face | iCBT of various sorts | Qualitative interviews | 11 | 37 (31-48) | 6/11 female (55%) | 10/11 psychologists (91%); 1//11 psychotherapist (9%) | Had worked with CBT both online and face-to-face | Therapists viewed face-to-face therapy as a stronger experience, and online therapy as offering more control over the workday. |
| Buti et al. 2013 | USA | Community treatment centres for alcohol and drug use disorders | Internet CBT with therapist support | Quantitative organisational and counsellor surveys. | 96 | 49.3 (24-73) | 76% female, 23.9% male | Counsellors | Drug and alcohol counsellors | The intentions of counsellors to use internet interventions was influenced by perceived social norms and not by attitudes. |
| Carper et al. 2013^1^ | USA | Center for Anxiety and Related Disorders (CARD) and an additional local outpatient treatment facility | Computer-based delivery of psychological treatments either as an adjunct to TAU or stand-alone | Quantitative survey | 26 | 29.8 (24-41) | 23 female, 3 male | Not reported. | Clinicians delivering outpatient treatment of anxiety and mood disorders | Clinicians were neutral toward online treatment, the lowest ratings were in relation to seeing or hearing about online treatment being used. |
| Dijksman et al. 2017 | The Netherlands | Practising Psychologists in Limburg, The Netherlands | Blended care – defined as online interventions combined with face to face contact | Mixed method survey - quantitative and qualitative. Delivery method not specified | 66 | 49 (27-83) | 24 male (36%), 42 female (64%) | Psychologists | Practising psychologists | Users of blended care were more positive than non-users. |
| Donovan et al. 2015 | Australia | Mental health workers from a variety of settings | Online programs with minimal (phone or email) or no therapist contact | Quantitative. Online survey with pre-post measures of an informational presentation. Both intervention and control conditions were included. | 124 | Most participants were in the following groups: 18-25 (16.1%), 26-30 (23.4%) and 31-35 (16.1%) | Not reported. | Psychologists, Counsellors, Social Workers, Nurses, Youth workers, Case workers, OTs, Guidance officers, Researchers and Others | Mental health workers | The attitudes of health workers to online treatment can be changed through educational interventions |
| Eichenberg et al. 2016^1^ | Austria, Germany,Switzerland | Psychotherapeutic settings - no details provided | Serious games defined as computer or video games with an educative component, used within a psychotherapeutic context | Quantitative online survey | 234 | 44 (no range reported) | 71.4% female | Not specified | Psychotherapists | Therapists can see serious games as a possible adjunct to face to face therapy. Therapeutic orientation had an impact on acceptance. |
| Folker et al. 2018 | Sweden. Norway, The Netherlands, Denmark, Scotland | Routine care settings - Field visits to sites | European supported iCBT programs operating in a routine care context targeting common mental health disorders | Multiple comparative case studies. Qualitative (interviews and focus groups) & observational (demonstrations of programs) | 9 managers, 15 key staff | not given | not given | Not reported. (Therapists said to be mostly psychologists or psychiatrists) | managers and therapists | Identified 4 challenges to implementation of iCBT including integration into care systems, recruitment of patients, working practice of therapists, and sustainability of services. |
| Friesen et al. 2014 | Canada | University online therapy clinic | Guided iCBT for depression, generalised anxiety disorder or panic disorder | Qualitative interviews | 12 | Not reported. | Not reported. | Post-graduate clinical psychology students | Mental health treatment using iCBT | Training in iCBT enhanced skills. Students reported challenges and benefits of iCBT. |
| Gellatly et al. 2017 | UK | Practitioners who participated in a multi-site RCT for low intensity interventions for OCD. | Supported computerised cognitive behaviour therapy for OCD. | Qualitative interviews | 20 | 34.1 (24-59) | 18 females, 2 males | Not reported. | Psychological Wellbeing Practitioners | Identified challenges and benefits of low intensity interventions for practitioners, clients and services. |
| Gun et al. 2011^1^ | Australia | Online treatment clinic website | Internet treatment for anxiety or depression with or without therapist support. | Quantitative survey | 456 | Not reported separately for health professional sample. | Not reported separately for health professional sample. | Not reported. | Not reported. | Health professionals wanted training and information about internet based treatments, as well as ethical and clinical guidance and clarification of legal issues. |
| Hadjistavropoulos et al. 2017 | Canada | Community mental health clinics | iCBT for depression and anxiety - online with email/phone support | Mixed methods - Online survey with open (qualitative) and closed-ended (quantitative) questions | 33 (22 therapists and 11 managers) | Not reported. | 29 females, 4 males | 10 psychologists, 16 social workers, 5 nurses, 1 administrator, 1 counsellor | Therapists and managers | Identified barriers and facilitators to iCBT in community mental health clinics. The strongest barrier was the inner setting including resource availability and the emphasis on face to face care. |
| Hadjistavropoulos et al. 2014^1^ | Canada | Online Therapy Unit for Service, Education and Research at the University of Regina (co-ordinates mental health services including iCBT) | CBT provided over the internet with structured assignments between modules. | Qualitative interviews | 11 | Not reported. | Not reported. | Psychologists and/or social workers. | Community mental health providers | Providers were positive about their experiences delivering iCBT, with the most common negative feedback relating to aspects of the CBT presented. |
| Ijzerman et al. 2019 | Netherlands | Psychologists in a medical (hospital) setting | Online CBT with therapist email support, sometimes offered as blended therapy (combined with face to face treatment) | Quantitative online survey | 107 | 40.5 (range not reported) | 92 females (86%), 15 men (14%) | 87 Psychologists (81.3%), 20 Other (18.7%) | Medical psychology in hospitals | Intentions to use iCBT were higher than actual use which was low. |
| Kivi et al. 2015 | Sweden | Primary care therapists participating in the PRIM-NET project | Internet delivered CBT with asynchronous guidance from a therapist. | Mixed methods – Quantitative survey and qualitative interviews | 11 for survey, 4 for interview | Not reported. | Interview only reported: 2 females, 2 males | For survey:  10 Registered psychologists, 3 Psychotherapists, 1 Psychotherapist/psychologist | Primary care therapists | Therapists supported the use of ICBT in primary care and identified several barriers to implementation. |
| Lovell et al. 2017^2^ | UK | Practitioners who participated in a multi-site RCT for low intensity interventions for OCD. | Supported computerised cognitive behaviour therapy for OCD. | Qualitative interviews | 20 | 34.1 (24-59) | 18 females (90%), 2 males (10%) | Not reported. | Psychological Wellbeing Practitioners | Identified challenges and benefits of low intensity interventions for practitioners, clients and services. |
| Middlemass et al. 2012^1^ | UK | Health professionals working with patients with sleep problems including insomnia | Computerised cognitive behaviour therapy for insomnia integrated with online communities or social networks | Qualitative interviews and focus groups | 23 | Not reported. | Not reported. | Not reported. | Working with consumers with sleep problems | The two main themes involved concern about trust and functionality. |
| Montero-Marin et al. 2015^1^ | Spain | Primary care | Internet treatment for depression either guided or unguided. | Qualitative (interviews and focus groups) | Interviews: 10  Focus groups: 12 | Participants were in the following groups: 20-40 years = 6, 41-60 years = 14, >60 years = 3 | 9 males, 14 females. | 11 family physicians and 12 managers | Primary care | Health professionals wanted a standardised program and were concerned about extra workload (physicians) and cost-effectiveness (managers). |
| Pierce et al. 2016 | USA | Members of the Association for Contextual Behavioural Science familiar with Acceptance and Commitment Therapy (ACT). | This study looked at ACT-related mobile apps. | Quantitative survey | 356 | 40.77 (no range) | 67.4% female | Not reported. | 92% were delivering psychological services including therapy, coaching, consulting, etc. | Health professionals are interested in apps for out of session skills practice and maintenance of gains but use and familiarity is low. |
| Schroder et al. 2017^1^ | Germany | Members of professional organisations | Stand alone or guided internet treatments for depression typically based on CBT. | Quantitative - online self-report survey | 428 psychotherapists | 49.2 (27-70) | 68.7% female, 31.3% male | Not reported | Licensed psychotherapists. | Psychodynamically oriented therapists were more negative toward online treatment. |
| Shalom et al. 2015 | Israel | Convenience sample of Israeli therapists from public and private practices | Blended therapy - face-to-face therapy that incorporates technology (email, chat, video conversations or self-help applications). | Survey | 87 | 41 (24-70) | 58 females, 29 males | 12 social workers, 41 clinical psychologists, 5 neuropsychologists, 19 educational psychologists, 1 medical psychologist, 9 psychotherapists | Therapists | Therapists were positive toward blended therapy and this did not vary by therapeutic orientation. |
| Sinclair et al. 2013 | Australia | Rural health professionals who were given a Quick Guide to mental health resources prior to interview | Mental health websites offering standalone or guided treatment. | Qualitative interviews in 2 Phases | 21 (13 in Phase 1, 8 in Phase 2) | Phase 1 = 47  Phase 2 = 52 | Phase 1 = 10 female, 3 male  Phase 2 = 3 female, 5 male | Phase 1 = 4 GPs, 9 mental health specialists  Phase 2 = 4 GPs, 4 mental health specialists | Rural clinicians including GPs, psychologists, psychiatrists and clinical social workers | Clinicians were optimistic about online mental health resources in the future but were cautious in referring patients to them. |
| Van der Vaart et al. 2014^1^ | The Netherlands | Three mental health care institutions interested in or offering  blended therapy. | Blended online and/or face-to-face sessions | Delphi consensus (including surveys and interviews) | 12 | 45 (28-60) | 8 female, 4 male | Not reported. | Therapists treating patients with depression | Therapists are positive about blended therapy but want to be able to tailor treatment to patients. |
| Wells et al. 2007 | USA | Mailed survey to random sample of mental health professionals who were members of 11 professional organisations. | Broadly defined as mental health services provided via a computer | Quantitative Survey 1 - 1 page screening survey to confirm eligibility Survey 2 - detailed survey via online link. | 2,098 | Over ¾ were over 40. | About 60% female | Social workers (22%), psychologists (41%), were the two largest groups. | Direct client treatment. | Participants were unlikely to provide online treatment and were concerned about confidentiality and liability issues. |
| Whitfield & Williams 2004 | UK | National survey in UK | No clear definition - computerized CBT | Quantitative survey | 329 | Not reported | Not reported | Not reported | CBT treatment. | The use of computerised self-help was rare. Participants wanted more information and training before they would use this modality. |
| Wilhelmsen et al. 2014 | Norway | General practice – clinicians had been trained to use MoodGYM. | Internet based CBT with clinician support | Qualitative interviews | 11 | 34-58 | 2 males, 9 females | General practitioner | Primary care | While positive toward iCBT GPs struggled to implement module follow-ups and tended to return to standard treatment. |

Notes:

1. This study included data from multiple sources, only data on health professionals is included in the table and the review.
2. Only Study 2 (pp.83-94) is included in the table as the rest was not relevant to our review question. Data from the same study is reported in Gellatly et al. 2017.
