# Supplementary material for: Implementing Web-Based Therapy in Routine Mental Health Care: Systematic Review of Health Professionals’ Perspectives
Source: J Med Internet Res. 2020 Jul 23;22(7):e17362. doi: 10.2196/17362 (PMC7413287; doi:10.2196/17362)
Supplement: Multimedia Appendix 3 [file jmir_v22i7e17362_app3.docx]

| **Source** | **Question/objective sufficiently described?** | **Study design evident and appropriate?** | **Method of subject/comparison group selection OR source of information/input variables described & appropriate?** | **Subject (and comparison group, if applicable) characteristics sufficiently described?** | **If interventional and random allocation was possible, was it described?** | **If interventional and blinding of investigators was possible, was it reported?** | **If interventional and blinding of subjects was possible, was it reported?** | **Outcome & (if applicable) exposure measure(s) well defined & robust to measurement/misclassification bias? Means of measurement reported?** | **Sample size appropriate?** | **Analytic methods described/justified & appropriate?** | **Some estimate of variance is reported for the main results?** | **Controlled for confounding?** | **Results reported in sufficient detail?** | **Conclusions supported by the results?** |
| --- | --- | --- | --- | --- | --- | --- | --- | --- | --- | --- | --- | --- | --- | --- |
| Becker & Jensen-Doss 2013 | 2 | 2 | 2 | 2 | N/A | N/A | N/A | 2 | 2 | 2 | 2 | N/A | 2 | 2 |
| Buti et al. 2013 | 0 | 2 | 1 | 2 | N/A | N/A | N/A | 1 | 2 | 2 | 2 | 2 | 2 | 2 |
| Carper et al. 2013 | 2 | 2 | 2 | 2 | N/A | N/A | N/A | 2 | 2 | 2 | 2 | N/A | 2 | 2 |
| Dijksman et al. 2017^1^ | 2 | 2 | N/A | 2 | N/A | N/A | N/A | 2 | 2 | 2 | 2 | N/A | 2 | 2 |
| Donovan et al. 2015 | 2 | 2 | 2 | 2 | 2 | N/A | N/A | 2 | 2 | 2 | 2 | 2 | 2 | 2 |
| Eichenberg et al. 2016 | 2 | 2 | 1 | 1 | N/A | N/A | N/A | 1 | 2 | 1 | 0 | N/A | 1 | 2 |
| Gun et al. 2011 | 2 | 2 | 2 | 2 | N/A | N/A | N/A | 1 | 2 | 1 | 2 | 0 | 2 | 2 |
| Hadjistavropoulos et al. 2017^1^ | 2 | 2 | 2 | 1 | N/A | N/A | N/A | 2 | 2 | 2 | 2 | N/A | 2 | 2 |
| Ijzerman et al. 2019 | 2 | 2 | 2 | 2 | N/A | N/A | N/A | 2 | 2 | 2 | 2 | N/A | 2 | 2 |
| Pierce et al. 2016 | 2 | 2 | 2 | 2 | N/A | N/A | N/A | 2 | 2 | 2 | 2 | N/A | 2 | 2 |
| Schroder et al. 2017 | 2 | 2 | 2 | 2 | N/A | N/A | N/A | 2 | 2 | 2 | 2 | N/A | 2 | 2 |
| Shalom et al. 2015 | 2 | 2 | 2 | 2 | N/A | N/A | N/A | 2 | 2 | 1 | 1 | N/A | 2 | 2 |
| Van der Vaart et al. 2014^1^ | 2 | 2 | 1 | 1 | N/A | N/A | N/A | 2 | 1 | 2 | N/A | N/A | 2 | 2 |
| Wells et al. 2007 | 2 | 2 | 2 | 2 | N/A | N/A | N/A | 2 | 2 | 1 | N/A | N/A | 2 | 2 |
| Whitfield & Williams 2004^1^ | 2 | 2 | 2 | 0 | N/A | N/A | N/A | 2 | 2 | 1 | 2 | N/A | 2 | 2 |

Notes:

1. This is a mixed method study and is included in all three tables.
