# Supplementary material for: Implementing Web-Based Therapy in Routine Mental Health Care: Systematic Review of Health Professionals’ Perspectives
Source: J Med Internet Res. 2020 Jul 23;22(7):e17362. doi: 10.2196/17362 (PMC7413287; doi:10.2196/17362)
Supplement: Multimedia Appendix 4 [file jmir_v22i7e17362_app4.docx]

| **Source** | **Question/objective sufficiently described?** | **Study design evident and appropriate?** | **Context for the study clear?** | **Connection to a theoretical framework / wider body of knowledge?** | **Sampling strategy described, relevant & justified?** | **Data collection methods clearly described & systematic?** | **Data analysis clearly described & systematic?** | **Use of verification procedure(s) to establish credibility?** | **Conclusions supported by the results?** | **Reflexivity of the account?** |
| --- | --- | --- | --- | --- | --- | --- | --- | --- | --- | --- |
| Advocat & Lindsay 2010 | 2 | 2 | 2 | 1 | 2 | 1 | 1 | 0 | 2 | 0 |
| Alberts et al. 2018 | 2 | 2 | 2 | 1 | 2 | 1 | 1 | 0 | 2 | 0 |
| Batka et al. 2016 | 2 | 2 | 2 | 1 | 2 | 1 | 1 | 0 | 2 | 0 |
| Bengtsson et al. 2015 | 2 | 2 | 2 | 1 | 2 | 1 | 1 | 0 | 2 | 0 |
| Dijksman et al. 2017^1^ | 2 | 2 | 2 | 1 | 2 | 1 | 1 | 0 | 2 | 0 |
| Folker et al. 2018 | 2 | 2 | 2 | 2 | 2 | 2 | 2 | 2 | 2 | 0 |
| Friesen et al. 2014 | 2 | 2 | 2 | 2 | 2 | 2 | 2 | 2 | 2 | 2 |
| Gellatly et al. 2017 | 2 | 2 | 2 | 2 | 2 | 2 | 2 | 2 | 2 | 1 |
| Hadjistavropoulos et al. 2017^1^ | 2 | 2 | 2 | 2 | 2 | 2 | 2 | 2 | 2 | 0 |
| Hadjistavropoulos et al. 2014 | 2 | 2 | 2 | 1 | 1 | 1 | 0 | 0 | 2 | 0 |
| Kivi et al. 2015 | 2 | 2 | 2 | 2 | 2 | 2 | 2 | 0 | 2 | 0 |
| Lovell et al. 2017 | 2 | 2 | 2 | 2 | 2 | 2 | 2 | 2 | 2 | 1 |
| Middlemass et al. 2012 | 2 | 2 | 2 | 2 | 1 | 1 | 2 | 2 | 2 | 0 |
| Montero-Marin et al. 2015 | 2 | 2 | 2 | 2 | 1 | 2 | 2 | 2 | 2 | 2 |
| Sinclair et al. 2013 | 2 | 2 | 2 | 2 | 2 | 2 | 2 | 2 | 2 | 0 |
| Van der Vaart et al. 2014^1^ | 1 | 2 | 2 | 1 | 2 | 2 | 2 | 1 | 2 | 0 |
| Whitfield & Williams 2004^1^ | 2 | 2 | 2 | 1 | 2 | 1 | 0 | 0 | 2 | 0 |
| Wilhelmsen et al. 2014 | 2 | 2 | 2 | 2 | 2 | 2 | 2 | 2 | 2 | 2 |

Notes:

1. This is a mixed method study and is included in all three tables.
