# Supplementary material for: Implementing Web-Based Therapy in Routine Mental Health Care: Systematic Review of Health Professionals’ Perspectives
Source: J Med Internet Res. 2020 Jul 23;22(7):e17362. doi: 10.2196/17362 (PMC7413287; doi:10.2196/17362)
Supplement: Multimedia Appendix 5 [file jmir_v22i7e17362_app5.docx]

| **Source** | **Justification of the mixed method design** | **Combination of qualitative & quantitative data collection-analysis techniques or procedures** | **Integration of qualitative & quantitative data or results** |
| --- | --- | --- | --- |
| Dijksman et al. 2017^1^ | 1 | 2 | 2 |
| Hadjistavropoulos et al. 2017^1^ | 2 | 2 | 2 |
| Van der Vaart et al. 2014^1^ | 2 | 2 | 2 |
| Whitfield & Williams 2004^1^ | 1 | 1 | 2 |

Notes:

1. This is a mixed method study and is included in all three tables.
